# Severe Covid-19 disease: rather AVDS than ARDS?

**DOI:** 10.1186/s13054-020-02972-w

**Published:** 2020-06-11

**Authors:** Yazine Mahjoub, Daniel Oscar Rodenstein, Vincent Jounieaux

**Affiliations:** 1 Cardiac Thoracic Vascular and Respiratory Intensive Care Unit, Amiens University Medical Centre, University Hospital Centre, Amiens, France; 2 Pneumology Department, Cliniques Universitaires Saint-Luc, Université Catholique de Louvain, Brussels, Belgium; 3 Pneumology Department, Amiens University Medical Centre, Amiens, France

Dear Editor,

We read with great interest the editorial by Gattinoni et al. describing two time-dependent chronological phenotypes of patients suffering from Covid-19 pneumonia [1]. We do not only agree about the role of vasoplegia accounting for hypoxemia but we also believe that this lung vascular disorder is the common denominator of Covid-19 pneumonia which is present at all stages of the disease, making useless to distinguish type 1 and type 2 Covid-19 ARDS.

In spontaneously breathing patients hospitalized for Covid-19 pneumonia, we have observed a severe hypoxemia despite Chest CT scans showing quite limited grass-ground like opacities. Because of the limited alveolar damage, such hypoxemia can be best explained by a low ventilation-to-perfusion (VA/Q) ratio where VA is preserved and Q is increased which we suggest to be related to an increase in pulmonary vascular flow and not to an inhibited hypoxic pulmonary vasoconstriction (supposing that Alveolar PO_2_ is preserved). Moreover, assuming that alveolar ventilation is conserved, compensatory hyperventilation rapidly leads to hypocapnia which is known to be a very powerful inhibitor of the hypoxic ventilatory response [2]. This may explain the surprising lack of dyspnea noted in these patients.

Some of these patients may worsen (extension of grass-ground like opacities, appearance of lung consolidations) requiring ICU transfer. These patients effectively present a non-typical ARDS with refractory hypoxemia, a slightly decreased lung compliance, and low lung recruitability (type 1 described by Gattinoni) and, in our experience, rarely show right ventricular dysfunction as in typical ARDS (personal data). In a recent post-mortem study on Covid-19 patients, Varga et al. found endothelial cell infection by SRAS-Cov2 and endotheliitis [3]. This might be explained by the presence of ACE-2 receptors on vascular endothelial cells [4]. All these findings suggest a specific pulmonary vascular disorder induced by SARS-CoV-2, leading us to consider it as an acute vascular distress syndrome (AVDS) more than an atypical ARDS. Nevertheless, some patients do evolve to a more typical ARDS (type 2 described by Gattinoni et al.) due to extensive lung consolidations. Finally, when such patients recover from Covid-19 pneumonia, we observe a return to normoxia despite the persistence and, sometimes the worsening, of lung consolidations compared to the early stage of ARDS.

To conclude, according to our findings and to recent publications, we hypothesize that Covid-19 patients, at any stages of their disease, are characterized by an increased pulmonary blood flow with intrapulmonary right to left shunt with limited alveolar injury.

## Authors’ response

Luciano Gattinoni, Davide Chiumello, Sandra Rossi

We thank Mahjoub and colleagues for their interest in our editorial [1]. At that time of writing, what we proposed was still a hypothesis based on sound physiological background. Indeed, severe hypoxemia associated with near normal lung mechanics and lung gas volume may be explained primarily by the V_A_/Q mismatch. Since then, there has been increasing evidence that the hypothesis was substantially correct, although the precise mechanism through which the virus assault to the endothelium [3] and its link to the pulmonary perfusion dysregulation is not clearly defined. We do not agree, however, with the statement that being COVID-19 disease primarily an endothelial disease, the distinction between two phenotypes that later we called L (type 1) and H (type 2) is no more necessary [5]. Indeed, the respiratory support should be sharply different between the two conditions. In addition, we must realize that, in some patients, regardless the treatment, the pneumonia may progress naturally, being characterized by diffused micro thrombosis in the lung vasculature and remarkable fibrosis [6]. Such a progression, which may be further worsened by the Patient Self Inflicted Lung Injury (if an adequate treatment is delayed), explains the unusual incidence of cysts, blebs, and pneumothorax in advanced state of type 2 patients.

## Data Availability

Not applicable.

## Abbreviation

AVDS: Acute vascular distress syndrome
